# Effects of silk fibroin in murine dry eye

**DOI:** 10.1038/srep44364

**Published:** 2017-03-10

**Authors:** Chae Eun Kim, Ji Hyun Lee, Yeung Kyu Yeon, Chan Hum Park, JaeWook Yang

**Affiliations:** 1 Ocular Neovascular Disease Research Center and T2B infrastructure Center for Ocular Disease, Inje University Busan Paik Hospital, Busan 614-735, Korea; 2 Nano-Bio Regenerative Medical Institute, College of Medicine, Hallym University, Chuncheon 200-702, Korea; 3 Departments of Otorhinolaryngology-Head and Neck Surgery, Chuncheon Sacred Heart Hospital, School of Medicine, Hallym University, Chuncheon 200-702, Korea; 4 Department of Ophthalmology, Inje University College of Medicine, Inje University Busan Paik Hospital, Busan 614-735, Korea

## Abstract

The study aimed to investigate the effects of silk fibroin in a mouse model of dry eye. The experimental dry eye mouse model was developed using more than twelve-weeks-old NOD.B10.*H2*^*b*^ mice exposing them to 30–40% ambient humidity and injecting them with scopolamine hydrobromide for 10 days. Tear production and corneal irregularity score were measured by the instillation of phosphate buffered saline or silk fibroin. Corneal detachment and conjunctival goblet cell density were observed by hematoxylin and eosin or periodic acid Schiff staining in the cornea or conjunctiva. The expression of inflammatory markers was detected by immunohistochemistry in the lacrimal gland. The silk group tear production was increased, and corneal smoothness was improved. The corneal epithelial cells and conjunctival goblet cells were recovered in the silk groups. The expression of inflammatory factors was inhibited in the lacrimal gland of the silk group. These results show that silk fibroin improved the cornea, conjunctiva, and lacrimal gland in the mouse model of dry eye. These findings suggest that silk fibroin has anti-inflammatory effects in the experimental models of dry eye.

Dry eye is a chronic ocular surface disease, and a serious dry eye will cause a visual disorder that affects quality of life^1^. Dry eye is compounded by destabilization of the tear film that leads to a decrease in tear production, which results from immoderate vaporization of tears on the ocular surface^2^. The continued imbalance of the tear film and dry eye condition will cause damage of the corneal epithelial cells and loss of the conjunctival goblet cells^3^,^4^,^5^,^6^. Long-term dry eye has been reported with increased expression of inflammation factors such as tumor necrosis factor-alpha (TNF-α), matrix metalloproteinase (MMP)-2, MMP-9, intercellular adhesion molecule-1 (ICAM-1), and vascular cell adhesion molecule-1 (VCAM-1) in the ocular surface^7^,^8^,^9^,^10^,^11^.

Dry eye is treated with artificial tears such as hyaluronic acid as well as with cyclosporine A, tetracyclines, macrolides, and omega-3 and omega-6 fatty acids^12^,^13^,^14^,^15^,^16^,^17^,^18^,^19^,^20^,^21^. However, this treatment is unsuitable for long-term therapy because it may induce critical side effects such as high blood pressure, glaucoma, cataract, and infection^22^. Hence, it is essential to discover new therapeutic agents with excellent efficacy for dry eye.

Silk fibroin is a natural protein produced by *Bombyx mori*. It has been widely used as a scaffold of biomaterials for tissue engineering and regeneration^23^,^24^,^25^,^26^. The main component of the silk fibroin protein has been reported to be the perfect substrate in a variety of cells for proliferation and adhesion. Silk fibroin has biodegradability, hemostatic properties, non-toxic low antigenic properties, and non-inflammatory properties in the biomedical field^27^,^28^,^29^,^30^. Silk fibroin is used as a surgical suture material, as well as wound dressing, drug delivery system, and contact lenses; it is used in various forms, such as a gel film and powder solution^31^,^32^,^33^. In addition, efficacy of silk fibroin was reported through the epithelial, limbal epithelial, limbal mesenchymal stromal, and endothelial in the cornea, and pigment epithelial cells in the retina^34^,^35^,^36^,^37^,^38^,^39^,^40^,^41^,^42^. However, there has been no report on the efficacy of silk fibroin in dry eye.

In this study, we investigated the effects of silk fibroin solution in an experimental dry eye mouse model. We investigated changes in tear production, corneal irregularity score, corneal epithelial cell detachment, density of conjunctival goblet cells, and inflammatory factors in the lacrimal gland after instillation of silk fibroin in an experimental model of dry eye.

## Results

### Tear production changes of silk fibroin

**Figure 1:**
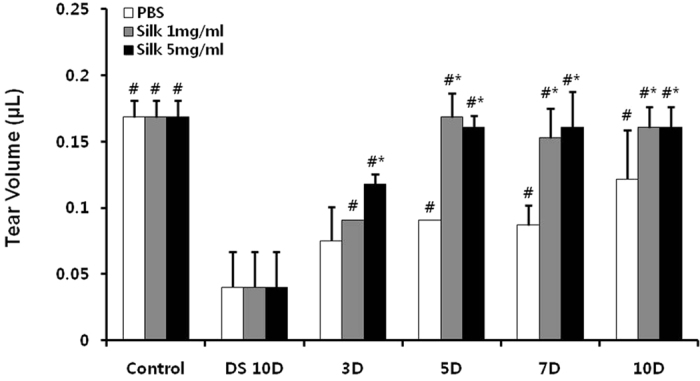
Effects of silk fibroin on tear production. Change in tear volume in the experimental dry eye model. *P <* *0.05 vs. the value in the PBS group. *^#^P *<* *0.05 vs. the corresponding value in the DS 10D group.

Compared with the PBS groups, tear production was increased in the silk groups (Fig. 1). Tear production was decreased to 76.2% in the desiccation stress for 10 days (DS 10D) group, compared with the control, and the PBS group exhibited increased tear production (3.0-fold) at 10 days, compared with the DS 10D group (*P* < 0.05). However, tear production was significantly increased by 4.0-fold in the 1 mg/mL and 5 mg/mL silk fibroin groups at 10 days, respectively, compared with the DS 10D group (*P* < 0.05). In addition, the tear volume in the 1 mg/mL and 5 mg/mL silk fibroin groups was significant increased to 1.3-fold at 10 days, compared with the PBS group (*P* < 0.05).

### Silk fibroin induced alteration in corneal smoothness

**Figure 2:**
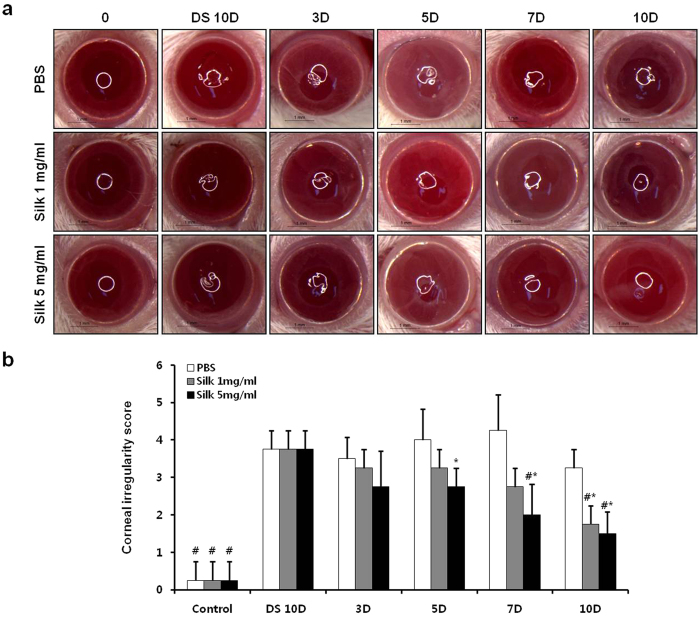
Effects of silk fibroin on corneal surface irregularities. (**a**) Corneal smoothness was imaged using a microscope. Scale bar = 1 mm. (**b**) Change of corneal irregularity scores. *P <* *0.05 vs. the value in the PBS group. *^#^P *<* *0.05 vs. the corresponding value in the DS 10D group.

After the desiccation stress did not change the shape of the white ring on the ocular surface in the PBS group. However, the shape of the white ring was improved on the ocular surface in the silk groups (Fig. 2a). The corneal irregularity scores were increased to 15-fold in the DS 10D group compared with the control, and a decrease to 13.3% in the PBS group was observed at 10 days compared with the DS 10D group (*P *<* *0.05) (Fig. 2b). The corneal irregularities were decreased to 53.3% and 60% in the 1 mg/mL and 5 mg/mL silk fibroin groups at 10 days, respectively, compared with the DS 10D group (*P* < 0.05). Moreover, the corneal irregularities were significantly decreased to 46.2% and 53.8% at 10 days in the 1 mg/mL and 5 mg/mL silk fibroin groups, respectively, compared with the PBS group (*P* < 0.05).

### Silk fibroin induced inhibition of epithelial cell detachment on the cornea

**Figure 3:**
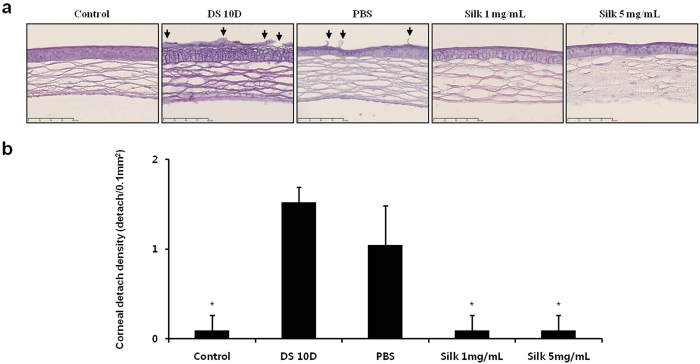
Effect of silk fibroin on detachment of corneal epithelial cells. (**a**) Hematoxylin and eosin staining. The *arrows* indicate the detached corneal epithelial cells. Scale bar = 100 μm. (**b**) The number of detached corneal epithelial cell. *P <* *0.05 vs. the corresponding value in the DS 10D group.

The corneal epithelial cells were stained with hematoxylin and eosin (H&E) (Fig. 3a). The detachment of corneal epithelial cells was increased to 16-fold in the DS 10D group compared with controls, and decreased to 93.8% in the silk groups, respectively, compared with the DS 10D group (*P* < 0.05) (Fig. 3b). Moreover, the detachment of corneal epithelial cells was decreased to 31.3% in the PBS group compared with the DS 10D group (*P* < 0.05). However, the number of detached corneal epithelial cells was significantly inhibited to 90.9% in the silk fibroin groups compared with the PBS group (*P* < 0.05).

### Silk fibroin induced recovery of goblet cells on the conjunctiva

**Figure 4:**
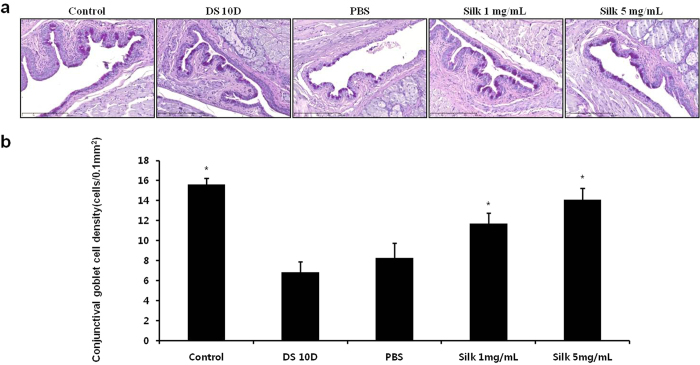
Effect of silk fibroin on conjunctival goblet cell densities. (**a**) Periodic acid Schiff staining. The *stained strong violet color* indicate the conjunctival goblet cells. Scale bar = 200 μm. (**b**) The number of conjunctival goblet cells. *P <* *0.05 vs. the value in the DS 10D group.

The goblet cells of the conjunctiva were stained with periodic acid Schiff (PAS) (Fig. 4a). The goblet cells densities of the conjunctiva decreased to 56.1% in the DS 10D group compared with the control (*P* < 0.05) (Fig. 4b). The goblet cell number of the conjunctiva increased to 1.7-fold and 2.1-fold in the 1 mg/mL and 5 mg/mL silk fibroin groups, respectively, compared with the DS 10D group (*P* < 0.05). However, the goblet cell densities of the conjunctiva increased to 1.2-fold in the PBS group compared with the DS 10D group (*P* < 0.05). Moreover, the densities of conjunctival goblet cells significantly increased to 1.4-fold and 1.7-fold at 10 days in the 1 mg/mL and 5 mg/mL silk fibroin groups, respectively, compared with PBS group (*P* < 0.05).

### Anti-inflammatory effects of silk fibroin

**Figure 5:**
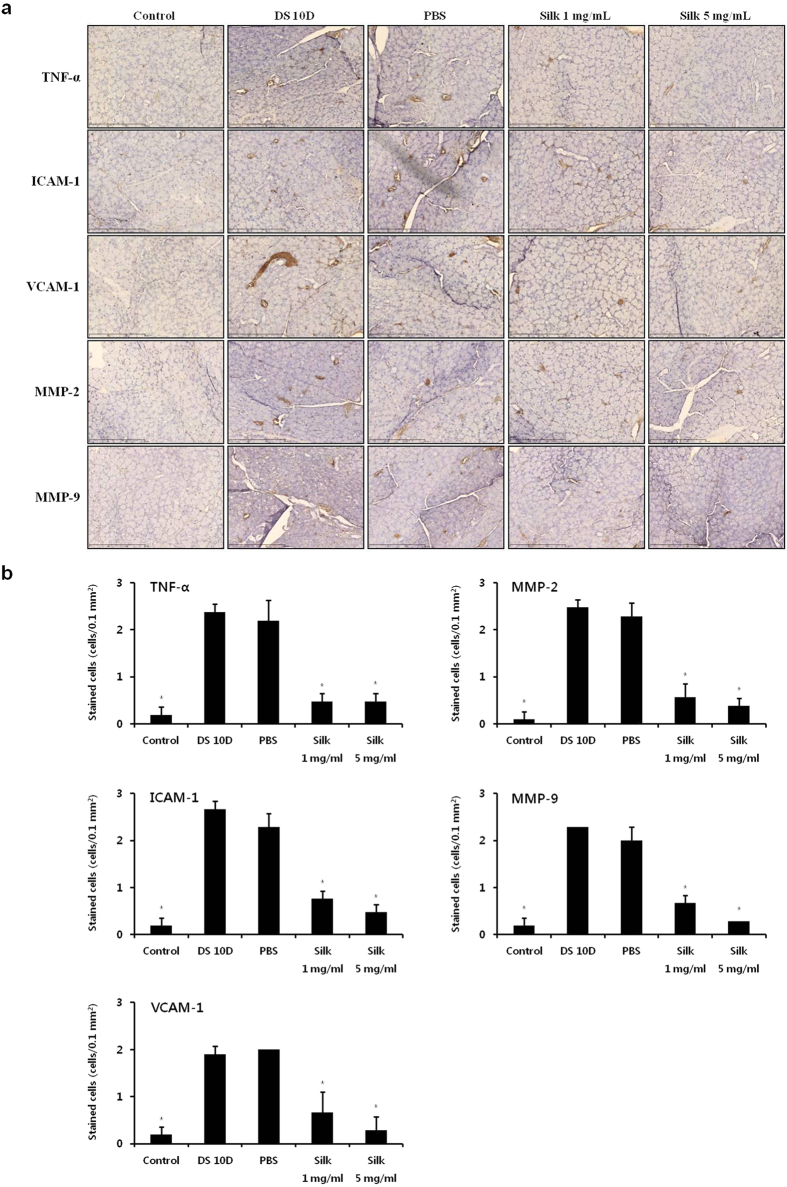
Effect of silk fibroin on inflammation. (**a**) Immunohistochemistry for inflammatory factors in the lacrimal glands of the mice. Scale bar = 300 μm. (b) The number of stained cells. *P <* *0.05 vs. the value in the DS 10D group.

Sections of the lacrimal gland were immunostained for TNF-α, MMP-2, MMP-9, ICAM-1, and VCAM-1 (Fig. 5a). All inflammatory factors were overexpressed in the DS 10D group compared with the controls (Fig. 5b). The number of stained TNF-α cells decreased by 80% in the silk groups, compared with the DS 10D group (*P* < 0.05). Staining for ICAM-1 was decreased by 71.4% and 82.1% in the silk groups, respectively, compared with the DS 10D group (*P* < 0.05). The stained VCAM-1 cells were inhibited by 65% and 85% in the silk groups, respectively, compared with the DS 10D group (*P* < 0.05). MMP-2 expression was decreased by 76.9% and 84.6% in the silk groups, respectively, compared with the DS 10D group (*P* < 0.05). The stained MMP-9 cells were inhibited by 70.8% and 87.5% in the silk groups, respectively, compared with the DS 10D group (*P* < 0.05). However, all inflammatory factors exhibited no significant decrease in the PBS group compared with the DS 10D group. Moreover, the expression of ICAM-1, VCAM-1, MMP-2 and MMP-9 was reduced by 37.5%, 57.1%, 33.3% and 57.1% in the 5 mg/mL silk fibroin groups, respectively, compared with the 1 mg/mL silk fibroin group (*P* < 0.05).

## Discussion

We confirmed the efficacy of silk fibroin in our experimental mouse model of dry eye. The silk fibroin exhibited increased tear production and reduced irregularity score of ocular surface in the dry eye. In addition, silk fibroin inhibited detachment of the corneal epithelial cells and recovered a number of conjunctival goblet cells in the dry eye model. Silk fibroin exhibited an anti-inflammatory effect by inhibiting the secretion of inflammatory factors by the lacrimal glands in the dry eye model.

Most dry eye treatments such as cyclosporine A, topical corticosteroids, tetracyclines, macrolides, and omega-3 and omega-6 fatty acids are known^12^,^13^,^14^,^15^,^16^,^17^,^18^,^19^,^20^,^21^. Cyclosporine and corticosteroids are anti-inflammatory agents that regulate the pro-inflammatory cytokines in the conjunctiva and lacrimal glands. Topical cyclosporine has been reported to improve tear production, decrease apoptosis of the corneal epithelial cells, and increase the number of conjunctival goblet cells through the inhibition of T-cell activation. However, cyclosporine appears to cause eye pain, irritation, and soreness symptoms in patients with dry eye, and long-term use of corticosteroids leads to side effects^22^. Tetracyclines and macrolides are antibiotics, which do not have an observed direct anti-inflammatory effect in dry eye. In addition, omega-3 and omega-6 fatty acids are unsaturated fatty acids, which help to supplement the lipid layer of tear film.

In a previous study, we investigated the a study on the effects of a therapeutic candidate agent in dry eye; we observed the effects of quercetin and chondrocyte-derived extracellular matrix in murine dry eye^5^,^6^. Quercetin is one of the flavonoid known to have antioxidant and anti-inflammatory effects; it decreased the detached epithelial cells of the cornea, reduced the number of conjunctival goblet cells, and exhibited anti-inflammatory effects in the lacrimal gland^5^. Chondrocyte-derived extracellular matrix is a new therapeutic agent that has demonstrated significantly suppressed neovascularization in a rabbit model of alkaline burn rabbit model and a pterygium mouse model as well as reduced the induction of inflammatory factors from the cornea or conjunctiva of a mouse model of dry eye^6^,^43^,^44^.

Silk is known to be useful in slowing tissue ingrowth into the biomaterial scaffold *in vivo* and *in vitro*, because of the slow rates of degradation^27^,^28^,^29^,^30^. In addition, there has been active research on the various formulations and preparation methods of the silk^31^,^32^,^33^. Silk has been studied in a variety of fields, such as bone tissue reconstruction, fabrication of artificial skin, acute tympanic membrane perforation, scaffolds for artificial esophagus, and segmental defects of the zygomatic arch^45^,^46^,^47^,^48^,^49^,^50^. In particular, silk membranes are suitable as a substrate for the restoration of damaged ocular surfaces to promote cellular attachment and growth in human limbal epithelial cells^34^,^35^.

Dry eye has been reported to cause inflammation, as well as abnormality of the cornea and conjunctiva from destabilization of the tear film, such as the lipid layer, aqueous layer, and mucous layer on the ocular surface^3^,^4^,^5^,^6^,^7^,^8^,^9^,^10^,^11^. These results suggest that the silk fibroin recovered the aqueous layer of tear film through its anti-inflammatory effects in the lacrimal gland and improved the mucus layer of tear film by increasing the number of conjunctival goblet cells in our experimental mouse model of dry eye. In addition, this improved tear film inhibited corneal epithelial damage, contributing to increased tear volume and improvement of ocular surface irregularity by the dry eye. This indicates that the silk fibroin provided a good environment through tear film stabilization in the ocular surface of the dry eye, which resulted in the recovery of the cornea, conjunctiva, and lacrimal gland of the dry eye.

In conclusion, our study demonstrated that silk fibroin has multi-target therapeutic effects that regulate inflammation of the lacrimal gland as well as ameliorate corneal and conjunctival abnormalities such as decreased tear production, deterioration of ocular surface smoothness, detachment of corneal epithelial cells and reduction of number of conjunctival goblet cells in dry eye. Therefore, silk fibroin is a potential new drug with great efficacy for treating inflammation and stabilizating the tear film in dry eye disease.

## Methods

### Preparation of silk fibroin solution

Silk fibroin aqueous solution was derived from *B. mori* cocoons. The procedure for the preparation of a silk solution is normally comprises three steps. The first process is a degumming step. Cocoons were finely chopped and boiled in 0.02 M sodium carbonate (Na_2_CO_3_) for an hour to be degummed. The silk fibers were then washed with distilled water and dried. The extracted silk fibroin was then dissolved in a mixing solution (CaCl_2_: Ethanol: H_2_O = 1: 2: 8) at 98 °C for 1 hour, yielding a 15 W/V% solution. This solution was dialyzed in distilled water using a dialysis membrane (MWCO 12,000–14,000, Spectra/Por, Spectrum Labs, Rancho Dominguez, CA) for 3 days. The concentration of the silk fibroin solution was calculated to 6 wt%. In this study, final silk fibroin solution was diluted to 0.1% (1 mg/mL) and 0.5% (5 mg/mL). The silk fibroin solutions were then stored at 4 °C before use to avoid premature precipitation^51^.

### Animals

The NOD.B10.*H2*^*b*^ mice used in the experiment were purchased through Jackson Laboratory (Bar Harbor, ME). This study was progressed in accordance with the guidelines for the care and use of laboratory animals of the Inje University College of Medicine and the Association for Research in Vision and Ophthalmology statement. All experimental protocols were approved by the Institutional Animal Care and Use Committee of Inje University College of Medicine (Approval ID: 2014–029).

### Experimental procedures of murine dry eye

**Figure 6:**
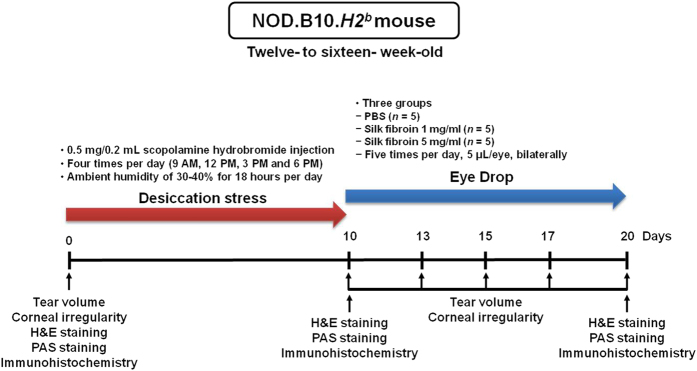
Schematic of experimental procedure. The experimental dry eye mouse model was developed as described in the Materials and Methods section. NOD.B10.*H2*^*b*^ mice received desiccation stress for 10 days and were then administered phosphate buffered saline or silk fibroin for 10 days.

Desiccation stress was created by low ambient humidity (30–40%) using an air draft from a fan for 18 hours per day, and 0.5 mg/0.2 mL hypodermic injection of scopolamine hydrobromide (Sigma-Aldrich, St. Louis, MO) into both hindquarters (one after the other) four times (9 AM, 12 PM, 3 PM, and 6 PM) per day for 10 days on more than 12-weeks-old male NOD.B10.*H2*^*b*^ mice^52^,^53^. After the desiccation stress for 10 days, the scopolamine hydrobromide injections were discontinued and the mice were placed in an environment of normal humidity and temperature. The eye drops, PBS and 1 mg/mL or 5 mg/mL silk fibroin were administered five times (9 AM, 11 AM, 1 PM, 3 PM, and 5 PM) per day for 10 days (Fig. 6).

The experiment was divided into the following groups: control (normal, *n *=* *5); DS 10D (untreated, desiccation stress for 10 days, *n *=* *5); PBS (*n *=* *5, 10 μL treated PBS in both eyes after the desiccation stress); silk fibroin 1 mg/mL (*n *=* *5, 10 μL treated 1 mg/mL silk fibroin in both eyes after the desiccation stress); and silk fibroin 5 mg/mL (*n *=* *5, 10 μL treated 5 mg/mL silk fibroin in both eyes after the desiccation stress). Mice in all groups were euthanized after the treatment period.

### Tear production measurements

Measurement of tear volume was performed using phenol red–impregnated cotton threads (Zone-Quick; Oasis, Glendora, CA), as previously described^54^. The threads were put in the lateral canthus of mice using a forceps for 20 seconds. The threads that absorb tears changed to red, and the red threads were evaluated in a millimeter through a microscope (SZX7; Olympus Corp., Tokyo, Japan). The tear volume was converted from millimeter to microliter compared with a standard curve^55^,^56^. The tear volume was measured within 2 hours of the scopolamine injection and within 1 hour of the PBS or silk fibroin administration, and evaluated by the average value from both eyes^5^,^6^.

### Measurement of the corneal smoothness and irregularity score

The corneal smoothness was measured after the euthanization of the mice, to obtain an image of the white ring under a fiber optic ring illuminator from a stereoscopic zoom microscope^6^. The smoothness of the cornea was measured within 2 hours of the scopolamine injection and within 1 hour of the PBS or silk fibroin administration, and evaluated by the average value from both eyes^5^,^6^. The corneal irregularity was evaluated by scoring the reflected distortion of the white ring of the epithelium of the cornea in the digital image, as previously described^15^. The irregularity scores of the cornea were evaluated according to the distorted quarters of the reflected ring, according to a five-point scale as follows: 0, no distortion; 1, 1/4 distortion; 2, 1/2 distortion; 3, 3/4 distortion; 4, 4/4 distortion; and 5, severe distortion and the shape of the ring is not recognized.

### Histologic experiment

The eyes and adnexa of each groups were surgically extraction, and then fixed by 10% formalin. The fixed tissues were embedded using paraffin and optimal cutting temperature compound (Tissue-Tek, Sakura Fine Technical Co., Ltd., Tokyo, Japan). The embedded tissues were sectioned to 6 μm, and then H&E and PAS staining was performed. The sections of each group were evaluated oven a range of 0.1 mm^2^ in the cornea and inferior fornices of the conjunctiva. The stained slides of each group were imaged through a Virtual Microscope (NanoZoomer 2.0 RS, Hamamatsu, Japan). The detachment of corneal epithelial cells and density of conjunctival goblet cells were evaluated as the average value of three non-consecutive cross-section slides from four mice per group.

### Immunohistochemistry

The eyes and adnexa were surgically excised, embedded in optimal cutting temperature compound, and flash frozen in liquid nitrogen. Six micrometer sections were excised with a cryostat. The sections were fixed with pre-cooled acetone for 5 minutes, and the primary antibodies to TNF-α (Abcam Inc., Cambridge, MA), MMP-2 (Abcam Inc., Cambridge, MA), MMP-9 (Lifespan Biosciences Inc., Seattle, WA), ICAM-1 (Bioss Inc., Woburn, MA), and VCAM-1 (Bioss Inc., Woburn, MA) were added and incubated for 1 hour at room temperature. After washing, the sections were incubated with secondary antibody (DAKO Corp, Glostrup, Denmark) for 30 minutes. Immunoreactions were visualized with diaminobenzidine chromogen, and the sections were counterstained with Mayer’s hematoxylin (Sigma-Aldrich) for 30 seconds at room temperature. Images of the sections were photographed with a Virtual Microscope (NanoZoomer 2.0 RS). The stained cells were evaluated oven a range of 0.1 mm^2^ in the lacrimal gland of each group.

### Statistical analysis

All data were analyzed using SPSS version 18.0 (SPSS, Chicago, IL) for Windows and reported as mean ± standard deviation. The comparative analysis of differences between groups were performed by two-way analyses of variance (with Tukey’s test), and statistical significance was indicated by *P *<* *0.05.

## Additional Information

**How to cite this article:** Kim, C. E. *et al*. Effects of silk fibroin in murine dry eye. *Sci. Rep.* **7**, 44364; doi: 10.1038/srep44364 (2017).

**Publisher's note:** Springer Nature remains neutral with regard to jurisdictional claims in published maps and institutional affiliations.
