# Corrigendum: Effects of silk fibroin in murine dry eye

**DOI:** 10.1038/srep46013

**Published:** 2017-04-07

**Authors:** Chae Eun Kim, Ji Hyun Lee, Yeung Kyu Yeon, Chan Hum Park, JaeWook Yang

Scientific Reports 7: Article number: 4436410.1038/srep44364; published online: 03 10 2017; updated: 04 07 2017

This Article contains an error in the Acknowledgements section:

“This study was supported by a grant from the Korea Healthcare Technology R&D Project of the Ministry of Health and Welfare Affairs, Republic of Korea (Grant No. HI15C1142)”.

should read:

“This study was supported by the Hallym University Research Fund and by a grant from the Korea Healthcare Technology R&D Project of the Ministry of Health and Welfare Affairs, Republic of Korea (Grant No. HI15C1142)”.

